# Integrative assessment of species-level genetic markers for the diagnosis of pathogenic *Leptospira*

**DOI:** 10.3389/fpubh.2026.1837984

**Published:** 2026-06-19

**Authors:** Luiza Aymée, Vinicius de Carvalho Moura, Myllena dos Santos Pereira, Walter Lilenbaum, Maria Isabel Nogueira Di Azevedo

**Affiliations:** 1 Laboratory of Veterinary Bacteriology, Biomedical Institute of Fluminense Federal University, Niteroi, Brazil; 2 Department of Microbiology, Immunology and Parasitology, Faculty of Medical Sciences, Rio de Janeiro State University, Rio de Janeiro, Brazil; 3 Laboratory of Investigation in Medical Microbiology, Institute of Microbiology, Federal University of Rio de Janeiro, Rio de Janeiro, Brazil; 4 National Reference Laboratory for Leptospirosis, Fiocruz, Rio de Janeiro, Brazil

**Keywords:** barcoding gap, BLAST, genetic marker, leptospirosis, phylogenetics

## Abstract

**Background:**

Leptospiral isolation and whole-genome sequencing are often unfeasible for genotyping; therefore, single-locus culture-independent sequencing is widely used. However, primer sets remain broadly applied without comprehensive quantitative or qualitative performance evaluation.

**Objective:**

This study integrated *in silico* primer performance, phylogenetic benchmarking, and barcoding gap analysis to identify *Leptospira* markers that best delimit species, support diagnostics, and enable standardized BLAST identity cut-off thresholds.

**Methods:**

An integrative analysis evaluated 10 primer pairs targeting seven loci: *secY* inner, *secY* outer, *lfb1, flaB, rpoB, gyrB,* and *lipL32*. New primers for 16S rRNA and *flaB* were designed and included in the framework. Primer hybridization and theoretical amplification were assessed *in silico* in genomes of five pathogenic *Leptospira* species. The best-performing markers were then tested for congruence with core-genome and full-*ppk* phylogenies. Global and species-specific barcoding gap analyses assessed genetic distances, and BLAST identity cut-offs were proposed for markers performing consistently across all steps.

**Results:**

Only five markers showed good theoretical amplification performance: 16S rRNA, *secY* outer, *flaB, rpoB,* and *lipL32*. However, only the phylogenies generated from *secY* outer, redesigned *flaB,* and *lipL32* were similar with core-genome and *ppk* trees. Barcoding analyses further showed that only *secY* outer and redesigned *flaB* could discriminate both species and intraspecific strains, with proposed BLAST identity cut-offs of 96 and 95%, respectively.

**Discussion:**

The present study extends barcoding gap analysis to bacterial marker evaluation within an integrative framework, an approach previously applied mainly to Metazoa. It also highlights an important limitation in the widespread use of genotyping markers that lack reliable *in silico* amplification performance and sufficient intra- and interspecific discriminatory power. Together, these findings support the combined use of *secY* outer and redesigned *flaB* for molecular surveillance of the main pathogenic leptospires, although further *in vitro* validation remains needed.

## 1 Introduction

Leptospirosis is an important, re-emerging bacterial disease with worldwide distribution, with marked endemicity in tropical and subtropical regions (1). As a classical One Health disease, it has an epidemiological cycle that depends on the interplay among animal reservoirs, bacterial environmental persistence, and subsequent human exposure (2, 3). Clinically, human leptospirosis ranges from an acute non-specific febrile illness to severe and life-threatening manifestations such as acute renal failure and jaundice (Weil’s disease), and/or pulmonary haemorrhage (1, 4). Globally, the disease was estimated to cause around 1 million cases and 60,000 human deaths per year (5). In animals, in addition to mortality, leptospirosis causes substantial economic losses, especially when it affects livestock production (2). The emerging environmental disasters further contribute to the spread of the disease and play a key role in the transmission of leptospires from animals to humans (6).

Leptospirosis is caused by spirochetes of the genus *Leptospira*, which currently comprises 74 species classified into four clades based on genome similarity (7, 8), reflecting differences in virulence, pathogenicity, and ecological niche. Clade P1 comprises the pathogenic leptospires, Clade P2 includes the intermediate species (3), and Clades S1 and S2 comprise the saprophytic leptospires (7). The pathogenic species most often associated with human and animal infections, and of particular relevance in a One Health context, are *L. interrogans, L. borgpetersenii*, *L. kirschneri,* and the emerging species *L. noguchii* and *L. santarosai,* all of which are classified within the highly pathogenic clade (P1+) (9–11).

The proper identification of circulating *Leptospira* strains is essential for understanding disease epidemiology, supporting effective outbreak surveillance, identifying host associations, and assessing transmission risks (3, 12). Robust genetic characterization, such as whole-genome sequencing (WGS), relies on bacterial culture, which is technically demanding and has low sensitivity due to the fastidious nature of leptospires, resulting in a limited number of isolates (13). In addition, the difficulty of maintaining leptospiral isolates often compromises the quantity and quality of genetic material required for a reliable WGS. To overcome those limitations, culture-independent genetic analyses have increasingly been performed directly on clinical samples for surveillance studies. In this context, Single Locus Sequence Typing (SLST) has played an important role in leptospiral surveillance (14). SLST is based on a single genetic marker for taxonomic identification and phylogenetic analysis and is particularly valuable because it can be applied directly to clinical samples (15). The most adopted markers are 16S rRNA/*rrs* (encoding the 16S ribosomal RNA), *lipL32* (encoding an outer membrane lipoprotein), *secY* (encoding a preprotein translocase subunit), *lfb1* (encoding a fibronectin-binding protein), *flaB* (encoding a flagellar filament core protein), *gyrB* (encoding the DNA gyrase subunit B), and *rpoB* (encoding the *β* subunit of RNA polymerase) (16, 17).

The wide range of genetic markers currently used in leptospiral genetic taxonomy compromises the construction of a homogeneous sequence database for surveillance (16). In addition to the lack of consensus on a universal marker for *Leptospira*, species-level cut-off values in BLAST-based comparisons are often defined arbitrarily across studies. Although this culture-independent approach has expanded leptospiral surveillance and enriched genetic databases, the inconsistent use of markers has raised concerns about whether they provide robust species-level discrimination or accurately reproduce the phylogenetic relationships inferred from phylogenomic approaches (7). Such inconsistency in marker choice and taxonomic resolution may lead to misclassification, ultimately compromising downstream analyses and limiting cross-study comparability.

Given these limitations, the genetic markers used for leptospiral taxonomy remain poorly characterized in quantitative terms. In this regard, the concept of a barcoding gap is particularly relevant, as it evaluates the existence of an interval between intraspecific and interspecific genetic distances, thereby supporting reliable species discrimination (18). Markers with good taxonomic resolution are expected to exhibit a clear barcoding gap (19). This approach has been widely applied to taxonomic inference in Metazoa using mitochondrial COI sequences (18). However, applications of this methodology to bacterial genes remain relatively limited and underexplored compared with its widespread use in Metazoa. In this context, the use of barcoding gap analysis to evaluate genetic targets in pathogenic *Leptospira* could be particularly useful for addressing the question of which markers are most suitable for taxonomic analysis.

To address the gap of a standardized and universally accepted genetic marker for species-level discrimination in pathogenic *Leptospira*, this study integrates primer performance, phylogenetic benchmarking against genomic gold standards, and barcoding gap analysis to determine which amplicon-based markers most accurately reflect species boundaries, are reliable for diagnostic applications, and allow the definition of standardized BLAST identity cut-off thresholds that can be applied in a universal and reproducible manner.

## 2 Materials and methods

### 2.1 Genome dataset retrieval and inclusion strategy

To maximize statistical power and evaluate primer performance under realistic diagnostic conditions, this study included all publicly available assembled genomes assigned to five major pathogenic *Leptospira* species (*L. interrogans*, *L. noguchii*, *L. kirschneri*, *L. borgpetersenii*, and *L. santarosai*) at the time of data retrieval (February 2026).

These species were selected because they represent the most frequently reported agents of human and animal leptospirosis across multiple epidemiological settings, encompassing broad host ranges and ecological contexts (14). They account for most molecular detections in clinical samples and represent the most extensively represented pathogenic taxa in public genomic repositories, providing a robust and statistically well-supported framework for evaluating barcode performance and species-level resolution.

As the primary objective was to assess the theoretical primer hybridization and amplification capacity and to evaluate the discriminatory power of each primer pair across the widest possible genomic diversity, no additional genome-quality filtering was applied beyond the taxonomic designations available in NCBI. All assembled genomes deposited in the NCBI RefSeq database, under the five species, were included.

This inclusive strategy was deliberately adopted to: (i) maximize sample size per species; (ii) capture intraspecific genomic diversity; (iii) simulate heterogeneous real-world datasets encountered in public repositories and diagnostic contexts; and (iv) avoid introducing bias through selective quality-based exclusion that could artificially inflate marker performance.

Available metadata were retrieved from NCBI and associated BioSample records and manually curated to standardize nomenclature.

### 2.2 Target gene selection and primer-based fragment extraction

Seven molecular markers commonly used for *Leptospira* detection and species identification were selected: 16S rRNA, *flaB*, *gyrB*, *lipL32*, *rpoB*, *secY*, and *lfb1*. Primer sequences and expected amplicon sizes are shown in Table 1. Additional short-amplicon primer pairs were designed for the 16S rRNA and *flaB* markers to enhance amplification efficiency in samples with low DNA concentration or partially degraded templates. Primer design was performed using Primer3 v2.6.1 and subsequently validated in Geneious Prime v2024.0.1 according to standard parameters, including melting temperature (Tm 58–62 °C), GC content (45–55%), and minimal potential for primer–dimer formation and secondary structures. Conserved regions were identified through MAFFT v.7 alignments of representative pathogenic species. Primer specificity was initially assessed using BLASTn against the NCBI RefSeq database to confirm genus-level exclusivity.

**Table 1:** Primers used for amplification of *Leptospira* spp. target genes, showing primer names, sequences (5′–3′), expected amplicon sizes (bp), and corresponding references.

Gene	Name	Primer sequence (5′-3′)	Size (bp)	Reference
16S rRNA	Lep1	GGCGGCGCGTCTTAAACATG	331	Mérien et al. (38)
	Lep 2	TTCCCCCCATTGAGCAAGATT		
	16S-F1	AACAACGCTTGCACCATACG	469	Present study
	16S-R2	CAAGTCAAGCGGAGTAGCAA		
*flaB*	L-flaB-F1	CTCACCGTTCTCTAAAGTTCAAC	772	Saito et al. (37)
	L-flaB-R1	TGAATTCGGTTTCATATTTGCC		
	flaB-F1a	CAGCAACGAAGATAGGCAGC	335	Present study
	flaB-R2a	CTCCCATATCCGCTCTCTGC		
*gyrB*	2For	TGAGCCAAGAAGAAACAAGCTACA	502	Slack et al. (28)
	504Rev	MATGGTTCCRCTTTCCGAAGA		
*lipL32*	LipL32-270-F	CGCTGAAATGGGAGTTCGTATGATT	423	Levett et al. (32)
	LipL32-692-R	CCAACAGATGCAACGAAAGATCCTTT		
*rpoB*	Lept 1900f	CCTCATGGGTTCCAACATGCA	600	La Scola et al. (39)
	Lept 2500r	CGCATCCTCRAAGTTGTAWCCTT		
*secY*	secY_outer_F	ATGCCGATCATTTTTGCTTC	554	Ahmed et al. (40)
	secY_outer_R	CCGTCCCTTAATTTTAGACTTCTTC		
	secY_inner_F	CCTCAGACGATTATTCAATGGTTATC	410	Grillová et al. (12)
	secY_inner_R	AGAAGAGAAGTTCCACCGAATG		
*lfb1*	LFB1-F	CATTCATGTTTCGAATCATTTCAAA	331	Merien et al. (29)
	LFB1-R	GGCCCAAGTTCCTTCTAAAAG		

Amplicon extraction was performed using SeqKit v2.13.0 (20) with the amplicon function, which identifies primer-binding sites within assembled genomes and extracts the intervening sequences. A conservative stringency condition was applied to approximate realistic PCR performance, allowing a maximum of two mismatches per primer (−m 2). This criterion was adopted to account for limited primer-template variation that may occur *in vitro* while maintaining conditions compatible with applications involving direct amplification from clinical samples.

For each genome and primer pair, successful amplification was recorded when both forward and reverse primers were detected in correct orientation and within the expected size range. Extracted amplicons were compiled into marker-specific FASTA files, and headers were standardized.

Amplicon quality was subsequently evaluated prior to downstream analyses. Sequences were retained only if they met the following criteria: (i) length within ±5 nucleotides of the expected amplicon size; and (ii) absence of internal stop codons for protein-coding genes. Length filtering and ambiguity screening were performed using SeqKit and custom scripts, and coding sequence integrity was assessed by translation in Geneious Prime v2024.0.1. Amplicons failing these quality thresholds were excluded.

Amplification success was calculated separately for each species as the proportion of genomes in which the primer pair generated a detectable in silico amplicon of the expected size and primer-binding configuration. The inclusion criteria for the integrated discriminatory analysis required primer pairs to achieve ≥90% amplification success in at least four of the five target species under at least one stringency condition. This threshold was empirically defined as a conservative operational criterion to prioritize markers with consistently high amplification performance across the major pathogenic *Leptospira* species while minimizing exclusion due to a limited number of potentially divergent or outlier genomes.

### 2.3 Phylogenetic reconstruction and comparison

Only genetic markers whose primer pairs achieved ≥90% amplification success in at least four of the five target species were retained for phylogenetic analyses. For each eligible marker, up to 25 sequences per species were selected, whenever available, to ensure balanced taxon sampling while capturing intraspecific diversity. Sequence selection prioritized: (i) serovars (when available); and (ii) sequences from diverse hosts, geographic regions, and studies, aiming to represent the broadest possible ecological and epidemiological variability. Multiple sequence alignments were generated using MAFFT v7.525 (21) employing an iterative local alignment strategy (--localpair --maxiterate 1,000), which is particularly suitable for partial gene fragments and amplicon datasets that may contain limited insertions and deletions. This approach performs 1,000 cycles of iterative refinement to improve alignment accuracy.

Phylogenetic trees were inferred under the Maximum Likelihood (ML) framework using IQ-TREE v2.3.4 (22). The best-fitting nucleotide substitution model for each alignment was determined using ModelFinder (23), as implemented in IQ-TREE (−m MF), with model selection based on the Bayesian Information Criterion (BIC). After model identification, final tree inference was conducted using the selected evolutionary model and 10,000 bootstrap replicates (-B 10000), with the ultrafast bootstrap approximation method (24), to ensure high statistical robustness.

For each marker, two phylogenetic reconstructions were generated. The first tree included 25 sequences (when possible) per species passing the inclusion filters, providing a comprehensive representation of marker-wide diversity. These larger datasets were qualitatively compared with a reference phylogeny based on 1,371 core orthologous genes proposed by Vincent et al. (7), which served as a genomic gold-standard framework for species-level delineation and interspecific backbone structure. The second tree was constructed using a representative dataset comprising five sequences per species to facilitate visualization and qualitative inspection of the topological structure. Within this representative framework, the full-length *ppk* gene was used as the gold standard, as it was proposed by Vincent et al. (7) as a potential surrogate for the leptospiral core genome. The complete *ppk* sequence was analyzed using the same phylogenetic criteria and served as a high-resolution internal benchmark for the comparative assessment of species clustering and interspecific relationships across the evaluated markers. This dual strategy enabled both exhaustive phylogenetic assessment and enhanced interpretability of tree topology, while allowing direct comparison of amplicon-based markers against both a core-genome phylogenetic framework and an independent full-length locus with strong discriminatory performance.

Taxonomic resolution was evaluated based on three complementary phylogenetic criteria applied to both comprehensive and representative trees, compared to the reference core-genome (7) and *ppk* gene phylogeny, respectively. Phylogenetic congruence was evaluated using a semi-quantitative comparative framework based on three criteria: (i) species monophyly, (ii) backbone topology congruence, and (iii) deep-node bootstrap support. Species monophyly was assessed according to the consistency of species-specific clustering and absence of intermixing among taxa. Backbone congruence was evaluated relative to the reference *ppk* and core-genome phylogenies, considering the preservation of the major interspecific branching structure. Deep-node support was classified according to bootstrap values of the principal internal nodes defining interspecific relationships, with values ≥90 considered strong, 70–89 moderate, and <70 weak support. Star ratings were assigned comparatively across markers as a visual synthesis of overall phylogenetic performance.

### 2.4 Barcode gap analysis

#### 2.4.1 Model selection and genetic distance estimation

All analyses were conducted in R (v4.5.2; R Core Team). Pairwise nucleotide genetic distances were calculated using the package ape (25). For each marker, the previously selected model was used to estimate pairwise distances in R. This approach ensured that evolutionary distances reflected the substitution process most appropriate for each dataset, minimizing model misspecification and improving comparability of divergence estimates across loci. The full-length *ppk* locus was used as a reference benchmark to enable comparative evaluation of divergence patterns and marker performance across loci.

Pairwise distances were computed using the function dist.dna() in ape, with pairwise deletion applied to handle missing data. Distances were subsequently partitioned into: (a) intraspecific distances (D_intra), including all pairwise comparisons among sequences assigned to the same species, and (b) interspecific distances (D_inter), comprising all pairwise comparisons between sequences belonging to different species. Expected numbers of pairwise comparisons were verified for each dataset to confirm internal consistency and absence of duplication or mislabeling.

#### 2.4.2 Global (worst-case) barcode gap assessment

Barcode gap analysis followed the conceptual framework proposed by Zhang et al. (26). The primary level of evaluation was the global worst-case gap, designed to determine whether a universal species-level similarity threshold could be applied across the five target pathogenic *Leptospira* species included in this study.

For each locus, the maximum intraspecific distance observed across all species (max_intra) and the minimum interspecific distance observed across all species (min_inter) were identified. The strict global barcode gap was defined as:


$$\mathrm{Ga}p_{\text{global}}=\min\;({D^{\mathrm{all}}}_{\text{inter}})-\max\;({D^{\mathrm{all}}}_{\text{intra}})$$

A positive global gap (min_inter > max_intra) indicates complete separation between the highest observed intraspecific divergence and the lowest observed interspecific divergence under worst-case conditions. Negative values indicate overlap.

Markers maintaining a positive, strict global gap were considered eligible for conservative species-level identification.

#### 2.4.3 Species-specific barcode gap assessment

Following global evaluation, a refined species-level barcode gap analysis was performed. For each species within each marker, the maximum intraspecific distance and the minimum interspecific distance (nearest-neighbor distance) were identified.

The species-specific barcode gap was calculated as:


$$\mathrm{Ga}p_{\text{species}}=\min\;(D_{\text{inter}})-\max\;(D_{\text{intra}})$$

Positive values indicate complete separation for that species, whereas zero or negative values indicate partial or complete overlap between intra- and interspecific divergence.

This hierarchical approach (global first, species-specific second) enabled the identification of loci with universal discriminatory capacity, as well as markers exhibiting taxon-dependent performance.

#### 2.4.4 Barcode gap visualization

Graphical visualization followed the framework of Zhang et al. (26) and was implemented in R using the package BarcodingR.

Aligned FASTA datasets were imported using ape, and global barcode gap distributions were generated using the function barcoding.gap() in BarcodingR, which computes all pairwise intra- and interspecific distances and displays them as overlaid frequency histograms.

For transparency and reproducibility, distance matrices were independently calculated using dist.dna() in ape, and summary statistics (minimum, first quartile, median, mean, third quartile, maximum) were extracted directly from the distance vectors.

Histograms were generated using either barcoding.gap() (BarcodingR) for standardized global visualization, or geom_histogram() in ggplot2 for customized layouts.

**Figure 1:**
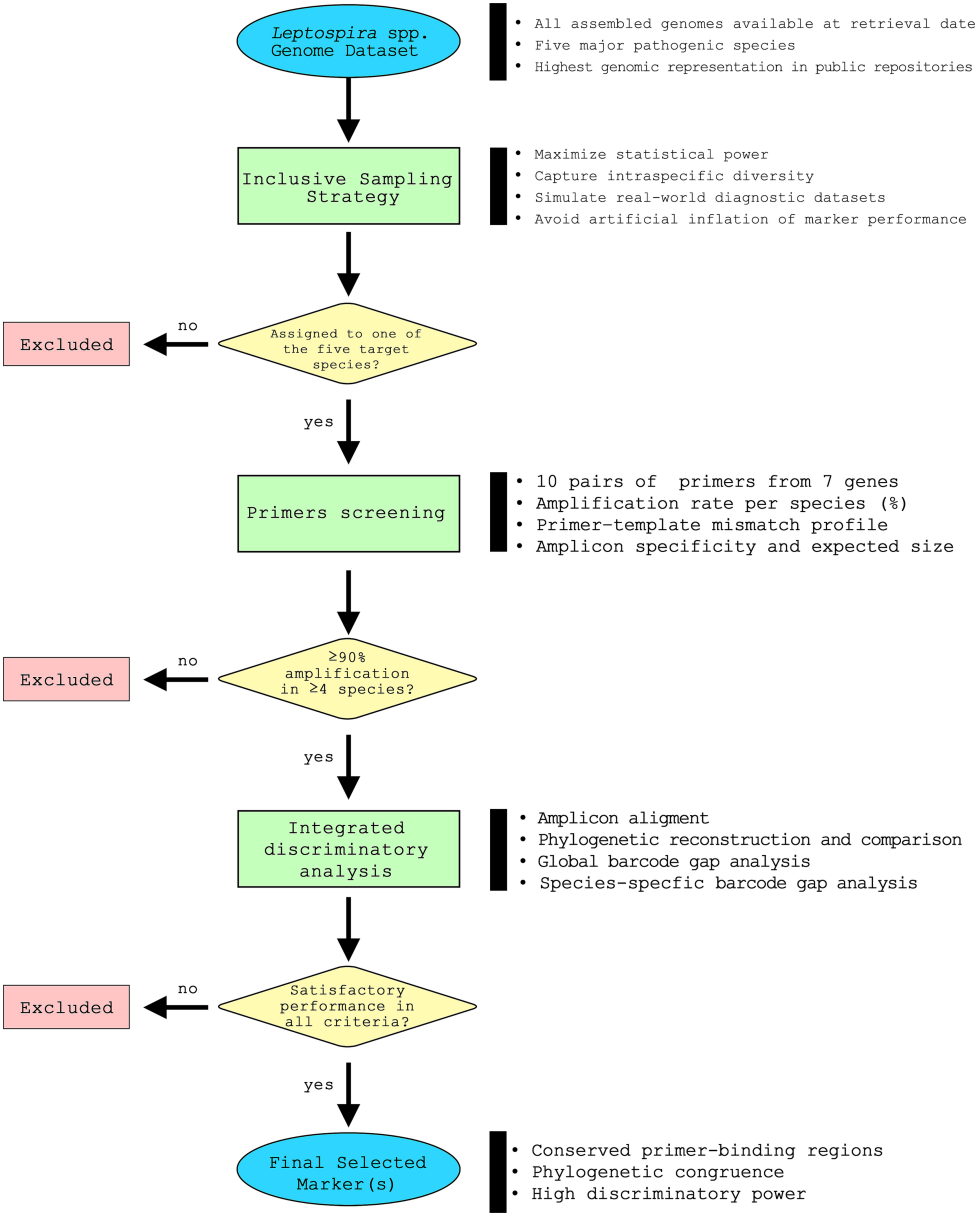
Methodological workflow for evaluating genetic markers in pathogenic *Leptospira*. All publicly available assembled genomes assigned to five major pathogenic species (*L. interrogans*, *L. noguchii*, *L. kirschneri*, *L. borgpetersenii*, and *L. santarosai*) were included, without additional quality filtering beyond taxonomic designation, to maximize statistical power and representation of intraspecific diversity. Primer pairs were screened for theoretical amplification performance, and those achieving ≥90% predicted amplification in at least four species under a conservative mismatch threshold of no more than two primer-template mismatches were retained. Selected markers were then evaluated using an integrated framework that combines phylogenetic reconstruction, global barcode gap assessment, and species-specific barcode gap analysis. Markers demonstrating consistent phylogenetic congruence and strong discriminatory power were retained as final candidates.

In all figures, the *x*-axis represents pairwise genetic distance, and the *y*-axis represents the absolute frequency of pairwise comparisons. Intraspecific distances were plotted in red and interspecific distances in blue. In species-specific barcode gap plots, regions of overlap between intra- and interspecific distributions were explicitly highlighted in gray to facilitate identification of problematic taxa. Species-level histograms were faceted by taxon to facilitate comparative assessment across markers. All figures were exported in high-resolution formats suitable for publication. The overall analytical workflow adopted in this study is summarized in Figure 1.

### 2.5 Empirical determination of species-level identity thresholds for BLAST-based identification

For genetic markers that demonstrated broad and stable *in silico* amplification across target species, phylogenetic congruence with established species-level relationships, and high discriminatory power as evidenced by a clear barcoding gap, species-level identity thresholds were empirically determined to support downstream BLAST-based taxonomic assignment.

For these markers, all pairwise intraspecific genetic distances were compiled across all species included in the dataset. The maximum observed intraspecific distance (d_max,intra) was identified under the best-fitting nucleotide substitution model previously selected for that marker. This value represents the upper bound of within-species genetic variation under the most divergent scenario observed.

Assuming high query coverage (i.e., near-complete alignment of the amplified fragment), sequence identity was approximated as:


Identity≈1–d

A conservative BLAST identity cutoff was then defined as:


$$\text{Cutof}f_{\text{BLAST}}=(1-d_{\max,\text{intra}})\times 100$$

where the resulting percentage value was rounded upward to the nearest integer to minimize the risk of false-positive species assignments.

This strategy ensures that any query sequence exceeding the proposed identity threshold falls within the empirically observed intraspecific variation range, while maintaining a safety margin against overlap with interspecific divergence.

## 3 Results

### 3.1 Genome dataset composition and metadata overview

A total of 893 assembled genomes assigned to the five-target pathogenic *Leptospira* species were retrieved from NCBI RefSeq database at the time of data collection. The dataset comprised: *L. interrogans*: 552 genomes; *L. borgpetersenii*: 189 genomes; *L. santarosai*: 67 genomes; *L. kirschneri*: 56 genomes; and *L. noguchii*: 29 genomes. Serovar information was available for 46.02% (411/893) of genomes. The inclusive retrieval strategy resulted in a dataset capturing extensive genomic, ecological, and geographic diversity within each of the five pathogenic species (Supplementary Table 1).

### 3.2 *In silico* amplification performance

Across the seven evaluated markers (16S rRNA, *flaB, gyrB, lipL32, rpoB, secY,* and *lfb1*), *in silico* amplification was assessed, allowing up to two mismatches per primer; amplification rates were from 0 to 100%, reflecting improved recovery under simulated realistic PCR conditions. Amplification success varied markedly among markers and species (Table 2). Under permissive mismatch conditions (≤2 mismatches per primer), distinct amplification profiles were observed, revealing substantial differences in primer universality and species coverage.

**Table 2:** *In silico* amplification success (%) of primer pairs under the adopted conservative mismatch threshold (≤2 mismatches per primer).

Gene	Primers (F/R)	Species				
		*L. int* (*n* = 552)	*L. borg* (*n* = 189)	*L. sant* (*n* = 67)	*L. kirs* (*n* = 56)	*L. nog* (*n* = 29)
16S rRNA	Lep1/Lep 2	92.4	99.5	91.0	92.9	86.2
	F1/R2	92.4	99.5	91.0	92.9	86.2
*lipL32*	270-F/692-R	99.6	100	100	100	100
*rpoB*	1900F/1900R	99.5	98.9	70.1	89.3	93.1
*flaB*	L-flaB-F1/L-flaB-R1	98.2	100	100	100	34.5
	F1a/R2a	98.6	100	100	100	100
*gyrB*	2For/504Rev	64.5	99.5	80.6	0	0
*rpoB*	1900F/2500R	99.5	98.9	70.1	89.3	93.1
*secY*	outer_F/outer_R	100	100	98.5	0	100
	inner_F/inner_R	99.3	0	0	0	24.1
*lfB*-1	LFB1-F/LFB1-R	98.9	0	0	100	100

*L. int*: *L. interrogans*; *L. borg*: *L. borgpetersenii*; *L. sant*: *L. santarosai*; *L. kirs*: *L. kirschneri*; *L. nog*: *L. noguchii*.

Among the evaluated loci, *lipL32* and *flaB* (F1a-R2a) demonstrated the most consistent cross-species amplification, achieving ≥98% recovery across all five pathogenic species. The *lipL32* gene region showed near-universal amplification (≥99.6% in *L. interrogans* and 100% in all other taxa), representing the most stable locus in terms of primer coverage.

The 16S rRNA primer sets exhibited high but slightly variable amplification (86.2–99.5%), with reduced recovery in *L. noguchii*. Similarly, *rpoB* performed well in most taxa but showed decreased amplification in *L. santarosai* (70.1%), suggesting lineage-specific primer mismatches.

In contrast, *secY*, *gyrB*, and *lfb1* displayed pronounced species-dependent amplification biases. Under the conservative mismatch threshold adopted in this study (≤2 mismatches per primer), the *secY* outer primer set did not amplify *L. kirschneri*, whereas the inner set showed limited amplification outside *L. interrogans*. The *gyrB* marker failed in *L. kirschneri* and *L. noguchii*, while *lfb1* exhibited amplification restricted to *L. interrogans*, *L. kirschneri*, and *L. noguchii*, with no recovery in *L. borgpetersenii* or *L. santarosai*.

Applying the ≥90% amplification success threshold, five primer pairs qualified for inclusion in the integrated discriminatory analysis. Markers meeting the inclusion criteria were: 16S rRNA/*rrs* (both primer sets: 16S-F1-R2 and Lep1-2), *lipL32* (LipL32-270-F-R), *rpoB* (270-F/ 692-R), *flaB* (F1a-R2a and L-flaB-F1-R1), and *secY* (outer F/R primer set). Amplicons of *secY* (inner primer set), *gyrB*, and *lfb1* were excluded.

### 3.3 Phylogenetic resolution and topological congruence

After model selection using ModelFinder under the Bayesian Information Criterion (BIC), the best-fit nucleotide substitution models were identified as follows: HKY + I for 16S rRNA/*rrs* (classic) and 16S rRNA/*rrs* (present study), K3Pu + I for *flaB* (classic), K3Pu + I + G4 for *flaB* (present study), TIM3 + F + G4 for *secY*_outer, and TN + F + 1 for *lipL32*. These models were subsequently implemented in all downstream analyses, including phylogenetic tree reconstruction and genetic distance calculations for barcode gap assessment.

**Figure 2:**
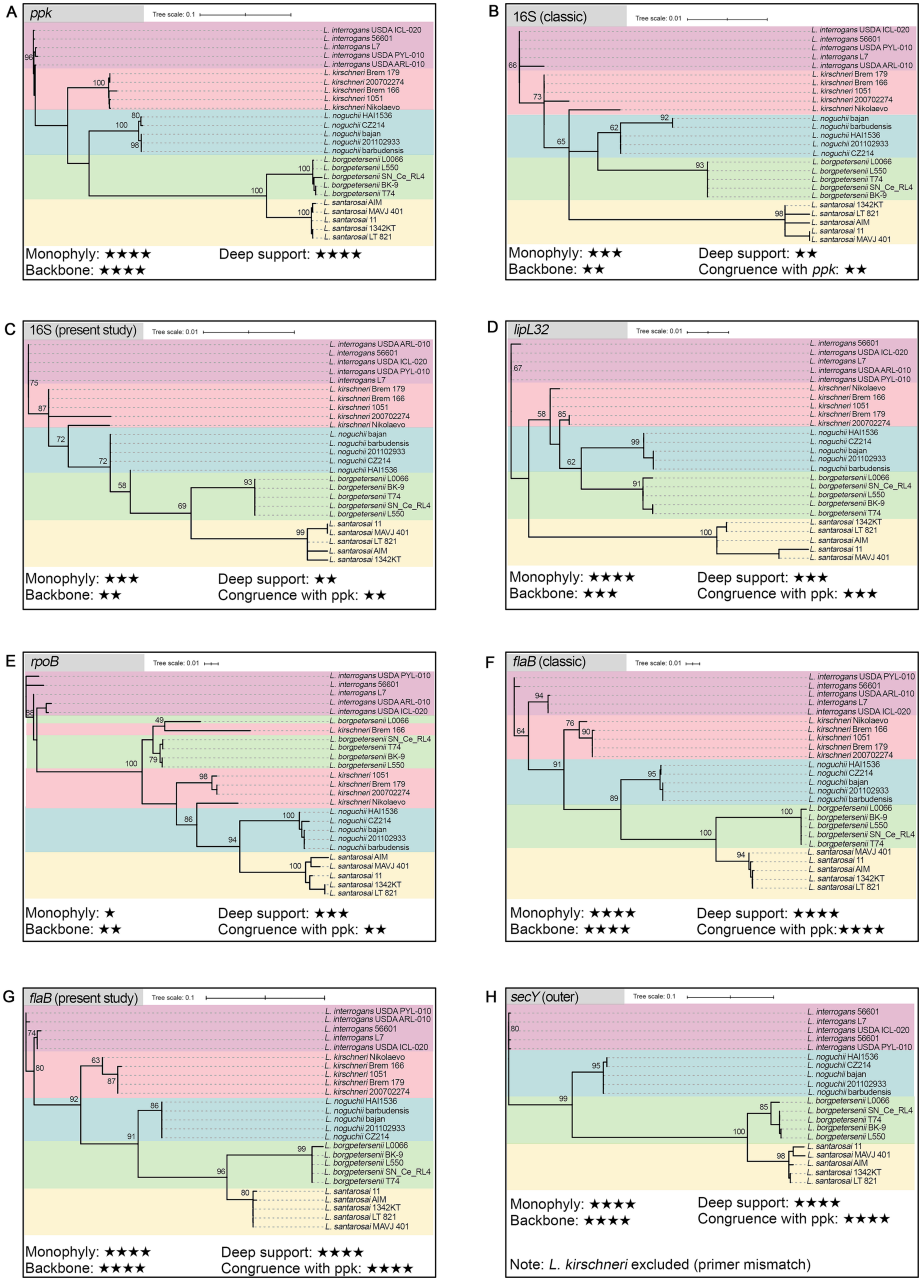
Phylogenetic reconstruction of candidate genetic markers for pathogenic *Leptospira*. Maximum likelihood (ML) trees inferred from representative datasets including five sequences per species are shown for each marker. Bootstrap values ≥50% are displayed at nodes. Panels **(A–H)** represent the evaluated genetic markers, as indicated within each subplot. Colored background shading indicates species clusters. For each marker, taxonomic resolution was evaluated based on species monophyly, backbone structure, deep-node support, and topological congruence with the ppk phylogeny. Star ratings summarize comparative performance across these criteria, ranging from low (★), moderate (★★), good (★★★), to high/excellent (★★★★).

Maximum likelihood phylogenetic reconstructions revealed marked differences in taxonomic resolution among evaluated markers. The representative trees (five sequences per species, Figure 2) were compared against the full-length *ppk* gene phylogeny, which served as the internal high-resolution benchmark, while the larger datasets (~25 sequences per species, Supplementary Figures) were qualitatively compared with the core-genome phylogeny proposed by Vincent et al. (7).

The full-length *ppk* gene recovered fully monophyletic species clusters with strong deep-node support and a stable interspecific backbone (Figure 2A). The topology was fully congruent with the core-genome phylogeny of Vincent et al. (7), validating its use as a high-resolution comparative framework. It is possible to observe a larger upper clade formed by *L. interrogans*, *L. kirschneri*, and *L. noguchii*, and a lower clade formed by *borgpetersenii* and *L. santarosai*, the latter being the most basal species.

Both 16S rRNA/*rrs* datasets (Figures 2B,C) recovered most species as distinct monophyletic clusters, but backbone resolution was consistently reduced compared with the *ppk* and core-genome reference phylogenies. Deep-node support values were generally lower, with several internal branches showing only weak-to-moderate bootstrap support. In both representative and expanded datasets, the relative positioning among *L. kirschneri, L. noguchii,* and *L. borgpetersenii* varied substantially compared with the reference topology. In addition, the expanded datasets revealed further backbone inconsistencies, including alternative branching arrangements and reduced support for deeper ancestral nodes (Supplementary Figures 1, 2).

The *lipL32* phylogeny recovered all five species as monophyletic clades but failed to reproduce the dichotomous backbone observed in *ppk*. Specifically, the basal sister-clade relationship between *L. borgpetersenii* and *L. santarosai*, strongly supported in the *ppk* tree (bootstrap 100), was not recovered by *lipL32*, which instead placed *L. santarosai* as the deepest-branching lineage and *L. borgpetersenii* as the next successive divergence in a pectinate arrangement. Deep internal nodes were only moderately supported (bootstrap 58–67), in contrast to the strong support observed in ppk (96–100). Expansion of the dataset genomes (Supplementary Figure 3) confirmed the strict monophyly of all five species, but also recovered a pectinate backbone topology, not congruent with the reference core genome tree (7).

The *rpoB* fragment (Figure 2E) showed limited phylogenetic resolution, failing to consistently recover reciprocal species monophyly. Notably, sequences assigned to *L. kirschneri* and *L. borgpetersenii* were intermingled across the tree, with some *L. kirschneri* lineages clustering within or adjacent to the *L. borgpetersenii* clade, indicating species non-monophyly (paraphyly/polyphyly) for these taxa. This pattern was also observed in the expanded dataset (Supplementary Figure 4), suggesting that the incongruence is not an artifact of reduced sampling. Although several internal nodes displayed moderate-to-high bootstrap support, the overall backbone topology remained distorted relative to the *ppk* benchmark and was likewise discordant with the core-genome reference phylogeny.

The classic *flaB* marker (Figure 2F) showed high congruence with *ppk*, with strong monophyly and well-supported deep nodes, although minor reductions in support values were observed relative to the full-length locus. On the other hand, in the expanded dataset, three *L. noguchii* genome sequences (GCF-000243575, GCF-000306195, GCF-000243535) were unexpectedly nested within the *L. borgpetersenii* clade (bootstrap 99), breaking the monophyly of *L. noguchii* (Supplementary Figure 5). Similarly, the short-amplicon *flaB* marker designed in this study (Figure 2G) demonstrated full recovery of species monophyly, strong deep-node support, and a backbone topology fully congruent with *ppk*. The branching order among major species-level clades mirrored both the *ppk* and core-genome frameworks. However, in the expanded dataset (Supplementary Figure 6), *L. borgpetersenii* was recovered as the deepest-branching lineage, not in congruency with *ppk* and core genome phylogenies.

The *secY* outer fragment (Figure 2H) recovered monophyletic clustering for all included taxa and exhibited strong backbone congruence with *ppk*. However, *L. kirschneri* was excluded due to primer mismatch, limiting its full cross-species assessment. Among the remaining species, interspecific relationships closely matched the reference topology, a pattern also observed in the expanded phylogeny (Supplementary Figure 7). Despite the exclusion of *L. kirschneri*, *secY* outer showed the highest overall phylogenetic performance among the evaluated markers, displaying maximal scores across all congruence criteria and the closest agreement with both the *ppk* and core-genome reference phylogenies.

With exception of *flaB* markers, the bigger trees (Supplementary Figure 1) largely mirrored the topological patterns observed in the representative trees. Markers that were congruent with *ppk* in the reduced dataset also showed congruence with the Vincent et al. (7) core-genome phylogeny under expanded sampling. Conversely, markers exhibiting incongruence in the representative trees remained discordant in the larger datasets.

### 3.4 Global barcode gap analysis across pathogenic *Leptospira* species

#### 3.4.1 Visual global barcode gap patterns

**Figure 3:**
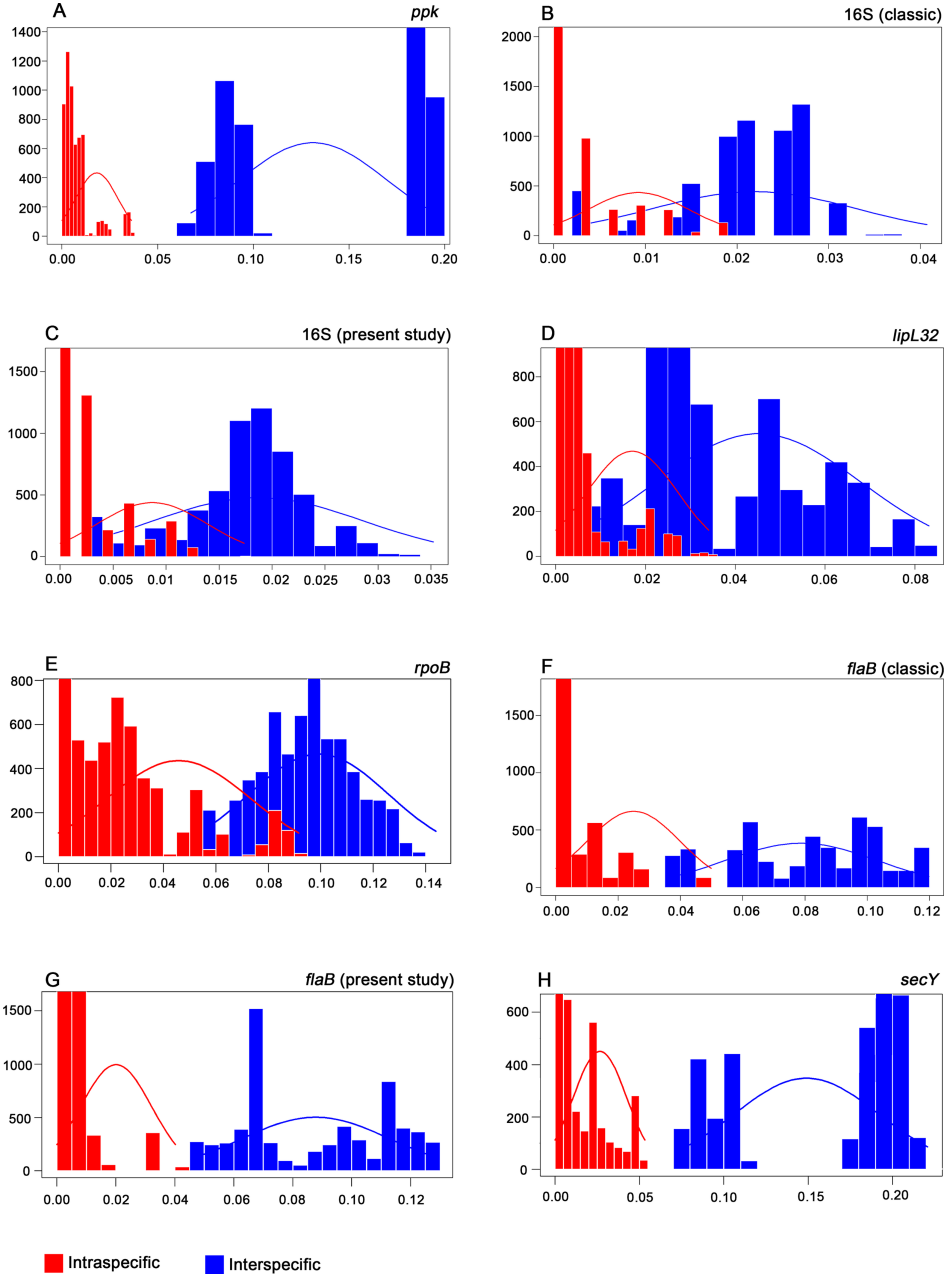
Global barcode gap analysis across genetic markers used for pathogenic *Leptospira* species. The x-axis represents genetic distance, while the y-axis represents the frequency of pairwise comparisons. Red bars correspond to intraspecific distances and blue bars to interspecific distances. Smoothed density curves are overlaid to illustrate distribution patterns. Panels correspond to: **(A)** *ppk*; **(B)** 16S rRNA (classic primers); **(C)** 16S rRNA (present study primers); **(D)** *lipL32*; **(E)** *rpoB*; **(F)** *flaB* (classic primers); **(G)** *flaB* (present study primers); and **(H)** *secY*. The degree of overlap between intra- and interspecific distributions reflects the discriminatory performance of each marker. A clear separation between maximum intraspecific and minimum interspecific distances indicates a positive global barcode gap and strong taxonomic resolution, whereas partial overlap suggests reduced species-level resolution.

Global barcode gap distributions revealed pronounced differences in discriminatory performance among markers (Figure 3). Frequency histograms of pairwise genetic distances demonstrated substantial variation in the degree of separation between intraspecific (red) and interspecific (blue) comparisons.

The full-length *ppk* gene exhibited complete separation between intra- and interspecific distributions (Figure 3A), with no visible overlap, indicating a strong and consistent global barcode gap. Similarly, the redesigned short *flaB* fragment (Figure 3G) and the *secY* fragment (Figure 3H) showed clear separation between distributions, reflecting robust global discriminatory performance.

In contrast, both 16S rRNA/*rrs* datasets (classic and redesigned fragments; Figures 3B,C) displayed extensive overlap between intraspecific and interspecific distances. The *lipL32* marker (Figure 3D) showed partial separation but with noticeable overlap, suggesting moderate resolution. The *rpoB* fragment (Figure 3E) exhibited pronounced overlap, consistent with unstable global discrimination. The classic *flaB* fragment (Figure 3F) showed intermediate performance, with partial but incomplete separation compared to the redesigned fragment.

Visual inspection thus suggested a clear hierarchy of marker performance, ranked according to their general discriminatory strength as follows: *ppk* ≥ *secY* (outer) ≈ redesigned *flaB* > classic *flaB* > *lipL32* > *rpoB* > 16S fragments.

#### 3.4.2 Quantitative global gap assessment (worst-case scenario)

To formally quantify these visual patterns, strict global barcode gaps were calculated for each marker across all five pathogenic species (Table 3). For each locus, the maximum intraspecific distance (max_intra) and the minimum interspecific distance (min_inter) were identified, and a worst-case global gap was computed; markers with Gap(d) > 0 (i.e., min_inter > max_intra) were considered to exhibit strict global separation.

**Table 3:** Comparative evaluation of currently available genetic markers for species-level discrimination based on global barcoding gap analysis.

Genetic marker	max_intra	min_inter	Gap (d)	Gap level	Species-level recommended?	Cutoff BLAST
16S rRNA (classic)	0.018	0.003	−0.015	No gap	No	—
16S rRNA (present study)	0.015	0.02	−0.013	No gap	No	—
*lipL32*	0.034	0.007	−0.027	No gap	No	—
*rpoB*	0.090	0.053	−0.037	No gap	No	—
*flaB* (classic)	0.050	0.038	−0.012	No gap	No	—
*flaB* (present study)	0.040	0.050	+0.010	Minimal gap	Yes	≥95%
*secY* (outer)	0.053	0.078	+0.025	Strong gap	Yes	≥96%

For each marker, the maximum intraspecific distance (max_intra) and the minimum interspecific distance (min_inter) were determined across all species. The global gap (Gap (d)) corresponds to the difference between min_inter and max_intra under a worst-case scenario. Gap levels were qualitatively classified as overlap (Gap < 0), minimal (0.01–0.019), or clear (≥ 0.02). Markers exhibiting a positive strict gap (min_inter > max_intra) were considered eligible for species-level assignment. When applicable, conservative BLAST identity cutoffs were derived from the highest observed intraspecific distance (identity ≈ 1 − d), assuming high query coverage.

Consistent with visual inspection (Figure 3), most loci failed to meet this criterion. Both 16S rRNA/*rrs* fragments showed negative global gaps (−0.015 and −0.013, respectively), indicating overlap between maximum intraspecific and minimum interspecific distances. Similarly, *lipL32* (−0.027), *rpoB* (−0.037), and classic *flaB* (−0.012) exhibited negative global gaps, confirming incomplete separation under worst-case conditions (Table 3).

In contrast, only the redesigned short *flaB* fragment (+0.010) and the *secY* fragment (+0.025) maintained positive strict global gaps. Although the redesigned *flaB* gap was classified as minimal (0.01–0.019), it still preserved universal separation across all species. The *secY* fragment exhibited the largest global gap, corresponding to a clear separation.

These quantitative results corroborate the global distribution patterns observed in Figure 3 and confirm that, among markers with available primer sets, only *flaB* (the present study fragment) and *secY* satisfy strict worst-case separation across all evaluated taxa.

### 3.5 Species-specific barcode gap analysis

**Figure 4:**
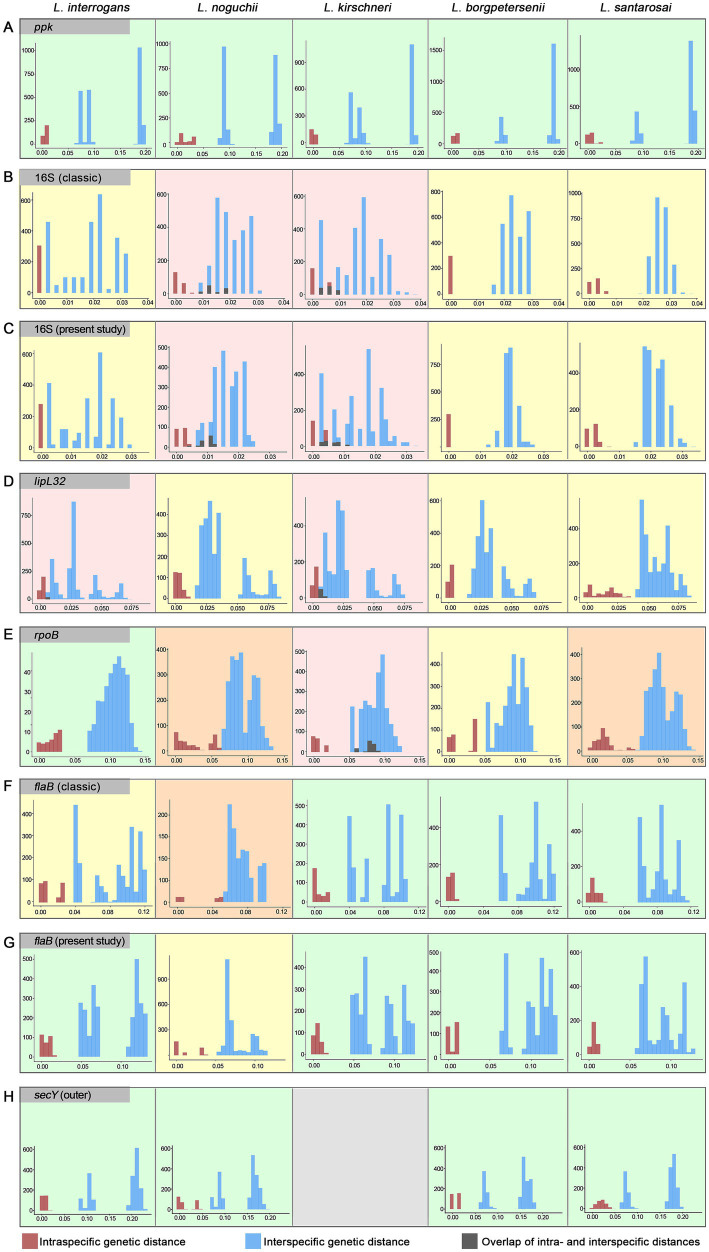
Barcoding gap analysis based on genetic distances across pathogenic *Leptospira* species and genetic markers. The *x*-axis shows genetic distances and the *y*-axis shows frequency. Histograms show pairwise distances for five species (*L. interrogans*, *L. noguchii*, *L. kirschneri*, *L. borgpetersenii*, and *L. santarosai*; columns) and eight markers [rows – **(A)** *ppk*; **(B)** 16S (classic); **(C)** 16S (present study); **(D)** *lipL32*; **(E)** *rpoB*; **(F)** *flaB* (classic); **(G)** *flaB* (present study); **(H)** *secY*]. All markers were analyzed according to the amplicon regions defined by primer sets, except for ppk, which was evaluated across its full-length sequence and used as a gold-standard reference for comparative purposes. As shown at the bottom of the figure, red bars indicate intraspecific distances, blue bars indicate interspecific distances, and gray bars overlap. Each panel background color summarizes the quantitative barcoding gap calculated as the difference between the minimum interspecific distance and the maximum intraspecific distance for that species-marker combination: red, negative gap (<0.00); orange, gap = 0.00; yellow, minimal gap (0.01–0.019); green, clear gap (≥0.02). For detailed inspection of distance distributions within each panel, zooming is recommended. *L. kirschneri* was not evaluated for *secY* due to lack of amplification under the adopted ≤2-mismatch criterion.

While the global analysis evaluates overall separation across the dataset, species-specific barcode gaps provide a more refined assessment of taxonomic resolution at the individual lineage level, enabling the identification of taxa that may present discriminatory challenges. In this analysis, the magnitude of the barcode gap varied substantially across genetic markers and species (Figure 4; Table 4). Gap values ranged from strongly positive (clear separation) to negative (distance overlap), revealing marked differences in discriminatory resolution. A complete compilation of all pairwise intra- and interspecific genetic distances calculated for each marker and species is provided in Supplementary Table 2.

**Table 4:** Quantitative barcoding gap values across pathogenic *Leptospira* species genetic markers.

Genetic marker	Gap size				
	*L. interrogans*	*L. noguchii*	*L. kirschneri*	*L. borgptersenii*	*L. santarosai*
*ppk*	0.060	0.046	0.057	0.073	0.060
16S (classic)	0.003	−0.009	−0.009	0.015	0.012
16S (present study)	0.002	−0.009	−0.013	0.013	0.009
*rpoB*	0.040	0.000	−0.037	0.016	0.000
*lipL32*	−0.002	0.010	−0.002	0.014	0.008
*flaB* (classic)	0.011	0.000	0.022	0.050	0.037
*flaB* (present study)	0.032	0.013	0.032	0.054	0.048
*secY* (outer)	0.067	0.029	NA*	0.055	0.025

Values represent the barcoding gap calculated as the difference between the minimum interspecific distance and the maximum intraspecific distance. Positive values indicate species-level separation, whereas negative values indicate overlap between intra- and interspecific distances. Cell background colors summarize gap magnitude: red, negative gap (<0.00; overlap); orange, gap = 0.00 (no separation); yellow, minimal gap (0.01–0.019); green, clear gap (≥0.02).

*NA: not applicable, as the secY primer set failed to amplify *L. kirschneri* under the conservative in silico amplification criteria adopted in this study (maximum of two mismatches).

The full-length *ppk* gene exhibited consistently large and positive barcode gaps across all five species, ranging from 0.046 (*L. noguchii*) to 0.073 (*L. borgpetersenii*) (Table 4). No overlap between intra- and interspecific distances was detected in any species (Figure 4A), confirming *ppk* as a high-resolution benchmark for species-level discrimination.

Both 16S rRNA/*rrs* datasets exhibited minimal or negative gap values. In 16S classic, gaps ranged from −0.009 (*L. noguchii*; *L. kirschneri*) to 0.015 (*L. borgpetersenii*). In the redesigned 16S fragment, gaps ranged from −0.013 (*L. kirschneri*) to 0.013 (*L. borgpetersenii*) (Table 4). Multiple species displayed negative or minimal gaps, reflecting substantial overlap between intra- and interspecific distances and limited taxonomic resolution (Figures 4B,C).

Gap values for *lipL32* were modest and variable. While *L. noguchii* (0.010), *L. borgpetersenii* (0.014), and *L. santarosai* (0.008) showed minimal positive gaps, negative values were observed for *L. interrogans* (−0.002) and *L. kirschneri* (−0.002), indicating overlap and incomplete species separation (Figure 4D; Table 3).

The *rpoB* fragment showed highly inconsistent performance. While *L. interrogans* exhibited a positive gap (0.040), *L. kirschneri* displayed a strongly negative value (−0.037), and zero gaps were observed for *L. noguchii* and *L. santarosai* (Figure 4E; Table 3). These results quantitatively corroborate the phylogenetic intermixing observed in Section 3.3.

The classic *flaB* fragment demonstrated positive gaps in most species, including strong separation in *L. borgpetersenii* (0.050) and *L. santarosai* (0.037). However, *L. noguchii* exhibited a gap of 0.00, indicating direct contact between intra- and interspecific distances and reduced discriminatory robustness (Figure 4F; Table 4).

The redesigned short *flaB* fragment displayed clear and stable barcode gaps across all species, with values ranging from 0.013 (*L. noguchii*) to 0.054 (*L. borgpetersenii*). All species showed positive gaps ≥0.02 except *L. noguchii* (0.013), which fell within the minimal gap range but remained positive. This marker closely approached the performance of *ppk* (Figure 4G; Table 4).

The *secY* outer fragment showed consistently positive, clear gaps across all evaluated species, ranging from 0.025 (*L. santarosai*) to 0.067 (*L. interrogans*). *L. kirschneri* was not evaluated due to primer mismatch. Gap magnitudes were comparable to those observed for *ppk* in several taxa (Figure 4H; Table 4).

Markers exhibiting negative or zero barcode gaps were the same loci that showed reduced species monophyly and backbone incongruence in phylogenetic reconstruction. Conversely, markers with consistently positive and large gaps (*ppk*, *flaB* new, *secY* outer) also demonstrated strong phylogenetic congruence.

### 3.6 Identity cutoff determination for BLAST-based assignment

Having established strict global separation for only two loci (Table 3), conservative BLAST identity thresholds were derived from the highest observed intraspecific distances for these markers. The redesigned *flaB* fragment yielded a conservative identity cutoff of ≥95%, while the *secY* fragment supported a threshold of ≥96% (Table 3).

Because only these two markers maintained strict worst-case separation, they were considered suitable for conservative BLAST-based species assignment. However, each presents locus-specific limitations: *secY* current primers do not amplify *L. kirschneri*, whereas redesigned *flaB* exhibits a minimal global gap and a certain degree of topological inconsistency in phylogenies inferred from larger genome datasets.

## 4 Discussion

Although whole-genome sequencing is currently the gold standard for bacterial genotyping, its application to *Leptospira* is limited by the difficulty and time required to culture this fastidious spirochete, as well as by resource constraints (12). Despite the recent reduction in sequencing costs, this approach remains financially demanding for low- and middle-income countries, which bear the greatest burden of leptospirosis (27). Therefore, culture-independent, amplicon-based approaches remain necessary for surveillance and leptospiral genotyping and are widely used across settings. The present study provides one of the first integrative and quantitative evaluations of commonly used single-locus genetic markers across five pathogenic *Leptospira* species associated with human and animal infections. It also offers a broad overview, curated for *L. interrogans, L. borgpetersenii, L. kirschneri, L. noguchii,* and *L. santarosai*, based on genomes from different geographic regions and hosts. These species are among the most frequently identified in clinical settings, but can also be isolated from the environment, reinforcing their relevance within a One Health framework (14).

Several findings raised concerns about the performance of genetic markers proposed in the literature and widely deposited in genetic databases. The first warning was that not all primer pairs hybridized with all five species, even under more permissive conditions allowing mismatches, suggesting limited theoretical sensitivity. This is particularly concerning because the present approach does not account for PCR inhibitors, which may further reduce assay sensitivity *in vitro*. Only five of the seven loci showed primer hybridization in more than 90% of genomes from at least four species. Two primer pairs for *gyrB* (28) and *lfb1* (29) loci did not meet the inclusion criteria to advance to the next stage of this integrative analysis. It is particularly concerning, especially regarding *lfb1*, because these primers have been used for diagnostic and sequencing purposes (30, 31). Because of this, the burden of leptospiral infections by these five *Leptospira* species may have been underestimated in studies that used these primers, unless, hypothetically, less stringent *in vitro* hybridization conditions than those applied here (i.e., allowing >2 mismatches) were achieved. In contrast, a positive finding was the consistent amplification of *lipL32* classical primers (32) in the pathogenic species, supporting its classification as a conserved region and reinforcing its suitability as a diagnostic screening target, as already widely recognized in both human and animal diagnostics (33). This marker has also been shown to amplify successfully *in vitro* across a wide range of studies (16).

Phylogenetic congruence analysis represented the second step of the integrative framework applied to markers that demonstrated adequate amplification performance in the initial screening. Evaluating whether phylogenies inferred from individual loci reproduce the topology and overall branching structure of the core-genome phylogeny is essential for assessing their suitability for molecular epidemiology. Among the evaluated markers, *secY* outer, both *flaB* primer pairs, and *lipL32* showed the highest overall congruence with the reference phylogenies, particularly regarding preservation of the major interspecific backbone and recovery of monophyletic species clusters in the representative datasets. However, some inconsistencies were observed in the expanded *flaB* datasets, particularly involving a small number of genomes that disrupted strict species-level clustering. These anomalies were not detected in the reduced dataset and likely reflect either interspecies recombination at the *flaB* locus or potential misclassification of certain genome assemblies. Nevertheless, the marker still demonstrated good overall resolution for genetic taxonomy and species-level phylogenetic inference across the major pathogenic *Leptospira* lineages.

Both 16S rRNA primer pairs and the *rpoB* phylogenies showed poor accuracy when compared with the reference trees. It is particularly concerning that two different 16S rRNA markers yielded similar poor results, further questioning the suitability of this locus for species-level discrimination in *Leptospira*. Although 16S rRNA sequencing is widely used in bacteriology, it has recognized limitations due to its high evolutionary conservation and low sequence variability, which restrict its discriminatory power among closely related taxa (34). Because of this conserved nature, 16S rRNA has often been used for *Leptospira* diagnosis; however, it does not reliably differentiate pathogenic from saprophytic species (16).

The third step of the framework consisted of a quantitative assessment of marker resolution based on barcoding gap analysis, which evaluated intra- versus interspecific genetic distances at both the global level (across the five species) and the species-specific level. Although this approach is classically applied to mitochondrial COI sequences in animals (18), it was successfully adapted here to leptospiral sequences, supporting its applicability to bacterial pathogens. The markers *lipL32*, 16S rRNA, and *rpoB* did not show adequate barcode gap sizes in either barcoding gap analysis, indicating limited genetic discriminatory power. In some cases, overlap between intra- and interspecific genetic distances was observed, which may lead to misclassification or failure in species discrimination. By contrast, *secY* outer and the redesigned *flaB* fragment retained positive global barcode gaps. However, *secY* outer could not be evaluated for *L. kirschneri* in the species-specific analysis, and *flaB* showed only a small gap for *L. noguchii*, suggesting limited discriminatory power among lineages within this species.

The present integrative analysis raises important concerns about the poor performance of some commonly used markers. This is particularly relevant because many sequences generated from these loci have been deposited in public databases and subsequently used in surveillance studies. In this context, the findings reinforce the importance of the present study, as the use of more suitable markers is essential for ensuring more reliable genetic characterization of circulating *Leptospira* strains and for supporting the development of a more homogeneous and comparable sequence database. Among the evaluated targets, two showed the most promising *in silico* performance: *secY* outer (554 bp) and the redesigned *flaB* (335 bp). The *secY* outer fragment has already been used *in vitro* as part of nested-PCR assays targeting *secY*; however, its performance as a standalone target still requires validation. Notably, many studies involving this locus have focused on the *secY* inner fragment, which is concerning, since *secY* inner fragment did not pass the primer hybridization step in the present analysis. Although *secY* outer performed well across all stages of the integrative framework, it is important to acknowledge a practical limitation observed under the conservative amplification criteria adopted in this study. Because our objective was to identify markers suitable for direct application to clinical samples, in silico amplification was evaluated using a maximum threshold of two primer-template mismatches. Under this criterion, the *secY* outer primer set did not recover amplicons from *L. kirschneri* genomes, preventing its evaluation for this species. This is particularly relevant because this pathogenic species has been involved in outbreaks of human leptospirosis (35) and identified in different animal hosts, including bats and rodents (36). Given the increasing reports of this species, the failure of the *secY* outer primers to amplify *L. kirschneri r*aises the question of whether new primer pairs should be designed to ensure broader coverage of pathogenic *Leptospira* species. Although this limitation is concerning, *secY* performed well in our framework, and numerous *secY* sequences have already been deposited in public databases, which facilitates sequence comparisons across studies. Therefore, while *secY* remains a useful marker, its use should be approached with caution and preferably complemented by additional loci to improve the reliability of species identification and phylogenetic inference.

The redesigned *flaB* marker also performed well and emerged as a promising alternative to overcome two important limitations of *secY* outer: its larger amplicon size, which may impair application in clinical samples, and its failure to amplify *L. kirschneri*. The new *flaB* primers generate a shorter amplicon than the original *flaB* assay (772 bp) (37), which may favor performance in field diagnostics and samples with low DNA quality. However, some limitations were observed in the species-specific barcoding analysis, particularly for *L. noguchii*, in which the marker displayed only a minimal barcode gap. At present, it remains unclear whether this pattern reflects an intrinsic limitation of the marker for resolving certain *L. noguchii* lineages or the comparatively limited genomic representation currently available for this species. Because only 29 *L. noguchii* genomes were available at the time of analysis, the statistical robustness of species-specific distance estimates for this taxon was likely lower than for more extensively represented species such as *L. interrogans*. Considering that *L. noguchii* is still comparatively underrepresented in public genomic databases, broader sampling will be necessary to determine whether the observed reduction in discriminatory power reflects genuine biological characteristics of the marker or current undersampling of the species diversity.

The combined use of *secY* outer and *flaB* is encouraged to increase the robustness of molecular surveillance and genetic characterization. However, it is important to note that the superior *in silico* performance of these markers does not necessarily translate into optimal *in vitro* performance, particularly in samples with low bacterial loads. Further *in vitro* validation is therefore required, especially in clinical and environmental samples, to assess parameters such as sensitivity, specificity, cross-reactivity, limit of detection, and sequencing success rate. Nevertheless, this redesigned *flaB* primer pair has already been applied in unpublished *in vitro* studies conducted by our group, where it showed good amplification performance. In addition, the present study established BLAST similarity cut-offs that may improve the reliability of species inference, indicating that species-level assignment should require at least 96% similarity for *secY* and 95% for *flaB*.

It is also important to highlight the full-length *ppk* gene, which was used as the gold standard for both the phylogenetic and barcoding gap analyses. This marker accurately reproduced the core-genome tree topology and showed positive gap sizes in both global and species-specific barcoding analyses. These findings reinforce the results of Vincent et al. (7) and support the idea that the full-length *ppk* gene can, to some extent, reflect genome-level relationships. Although *ppk* showed outstanding taxonomic resolution and the largest barcode gap among all evaluated markers, its performance must be interpreted within the framework adopted in this study. Despite its high discriminatory power, *ppk* was not included in the present framework analysis because it is not currently used in routine diagnostics or single-locus sequencing approaches. Moreover, no published and validated primer pairs are available for these applications, and the studies involving *ppk* have used the full-length gene (2,138 bp), which limits its applicability for PCR amplification directly from clinical samples, particularly those with low bacterial loads. Nevertheless, *ppk* remains a promising candidate marker and deserves further investigation due to its strong discriminatory potential.

Although the present study brought robust *in silico* results regarding the performance of markers used in single-locus sequencing for *Leptospira* characterization, some limitations must be acknowledged. Firstly, considering that this study relied on publicly available genomes with heterogeneous sequencing depth and assembly quality, these factors should be considered when reproducing the methodology, as fragmented or low-coverage assemblies may reduce target detection rates due to technical artifacts such as primer-binding regions interrupted by contig breaks rather than genuine biological sequence divergence. Moreover, it is important to highlight the unequal representation of species in the genomic dataset, ranging from 552 genomes for *L. interrogans* to only 29 for *L. noguchii*. This imbalance likely reflects both the limited availability of isolates and publicly available genomes, as well as the differing epidemiological relevance of each species. It is also important to emphasize that *in silico* amplification conditions do not account for PCR inhibitors or other factors that may affect *in vitro* performance and, therefore, may not be fully reproduced under laboratory conditions. In addition, all analyses were performed on sequences generated from specific primer pairs rather than full-length loci. Therefore, the performance evaluated herein should be interpreted as primer-pair performance, rather than as the intrinsic performance of the loci themselves.

In summary, the integrative framework adopted here proved essential, as reliance on a single analytical dimension, such as tree topology alone, could have overestimated marker performance. The pipeline presented here offers a reproducible approach that may be extended to the evaluation of species-discriminatory markers in other bacterial genera. For the genus *Leptospira*, this *in silico* study reinforces the value of *lipL32* as a diagnostic screening target and supports the combined use of *secY* outer and the redesigned *flaB* for sequencing, particularly in clinical samples where isolation and whole-genome sequencing are not feasible.

## Data Availability

The original contributions presented in the study are included in the article/Supplementary material, further inquiries can be directed to the corresponding author.
