# Supplementary figures and images for: Integrative assessment of species-level genetic markers for the diagnosis of pathogenic Leptospira
Source: Front Public Health. 2026 Jun 19;14:1837984. doi: 10.3389/fpubh.2026.1837984 (PMC13328341; doi:10.3389/fpubh.2026.1837984)

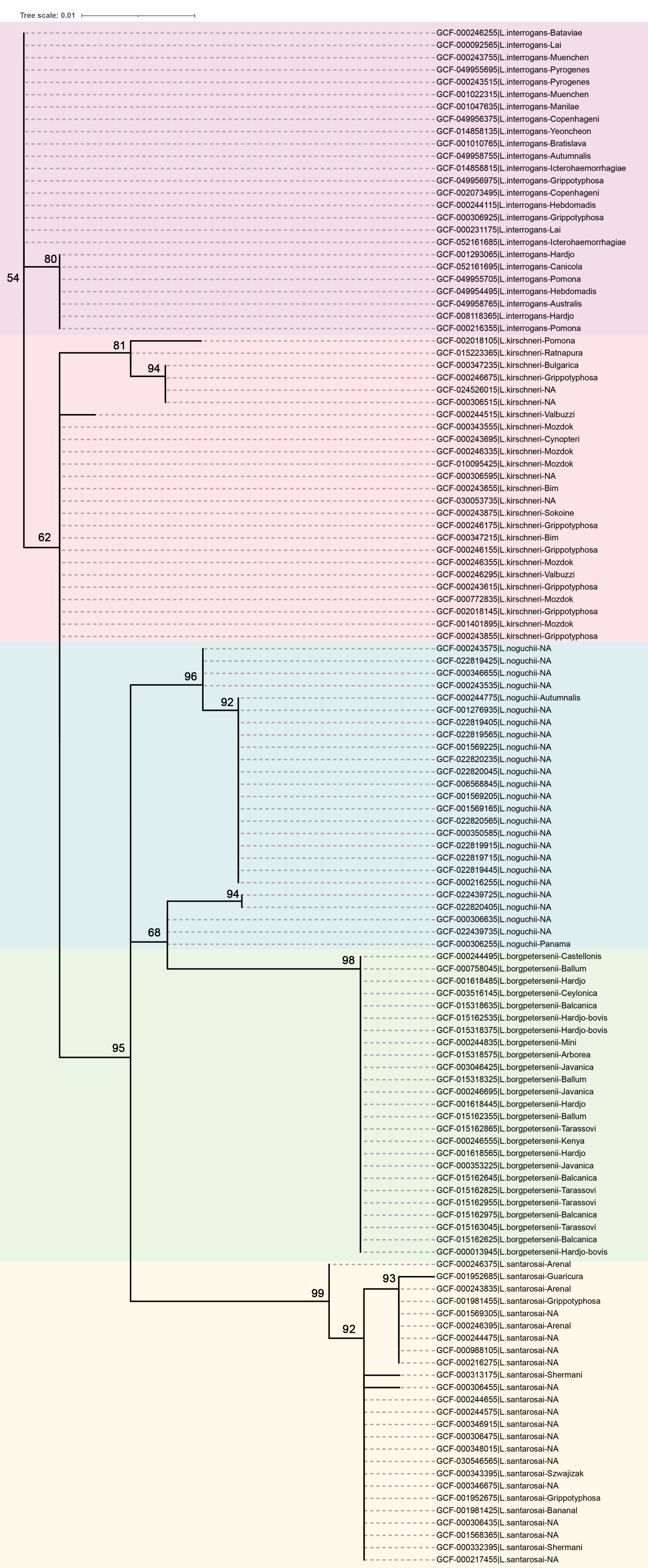

Supplement: Supplementary file 1 [file Image_1.JPEG]

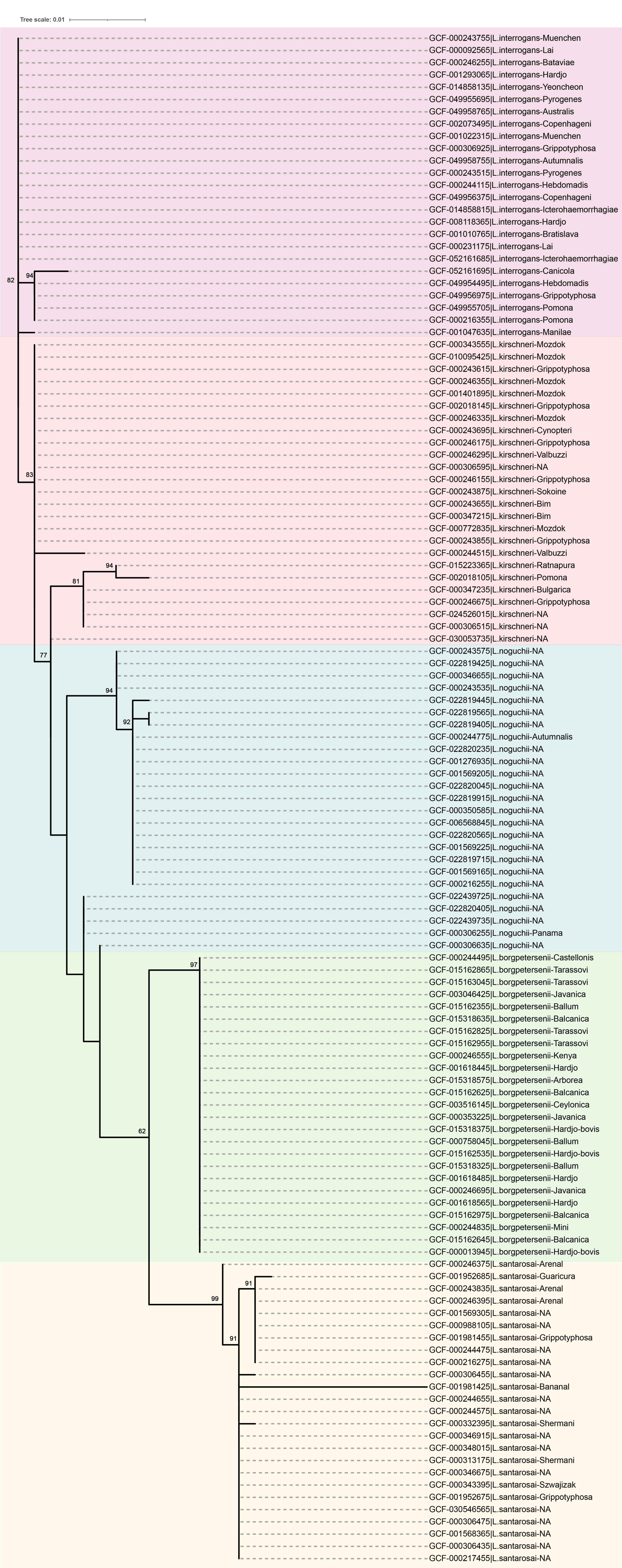

Supplement: Supplementary file 2 [file Image_2.JPEG]

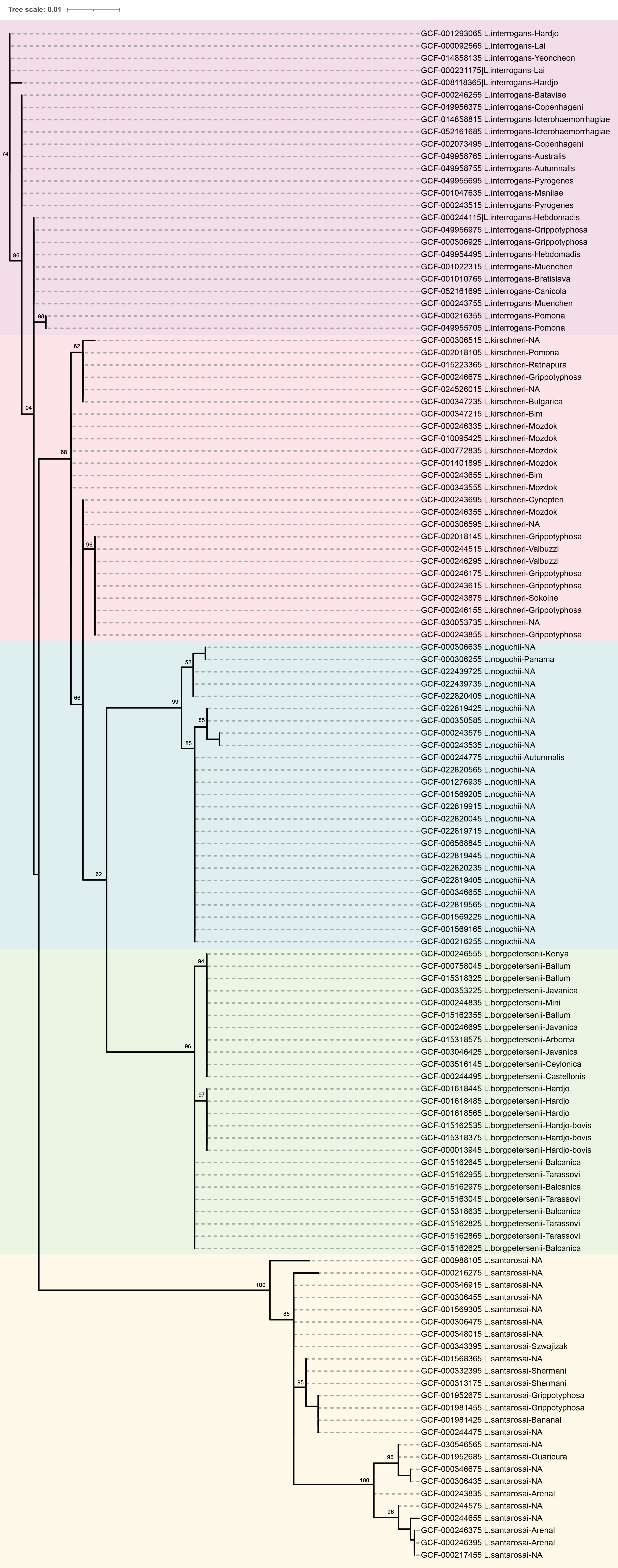

Supplement: Supplementary file 3 [file Image_3.JPEG]

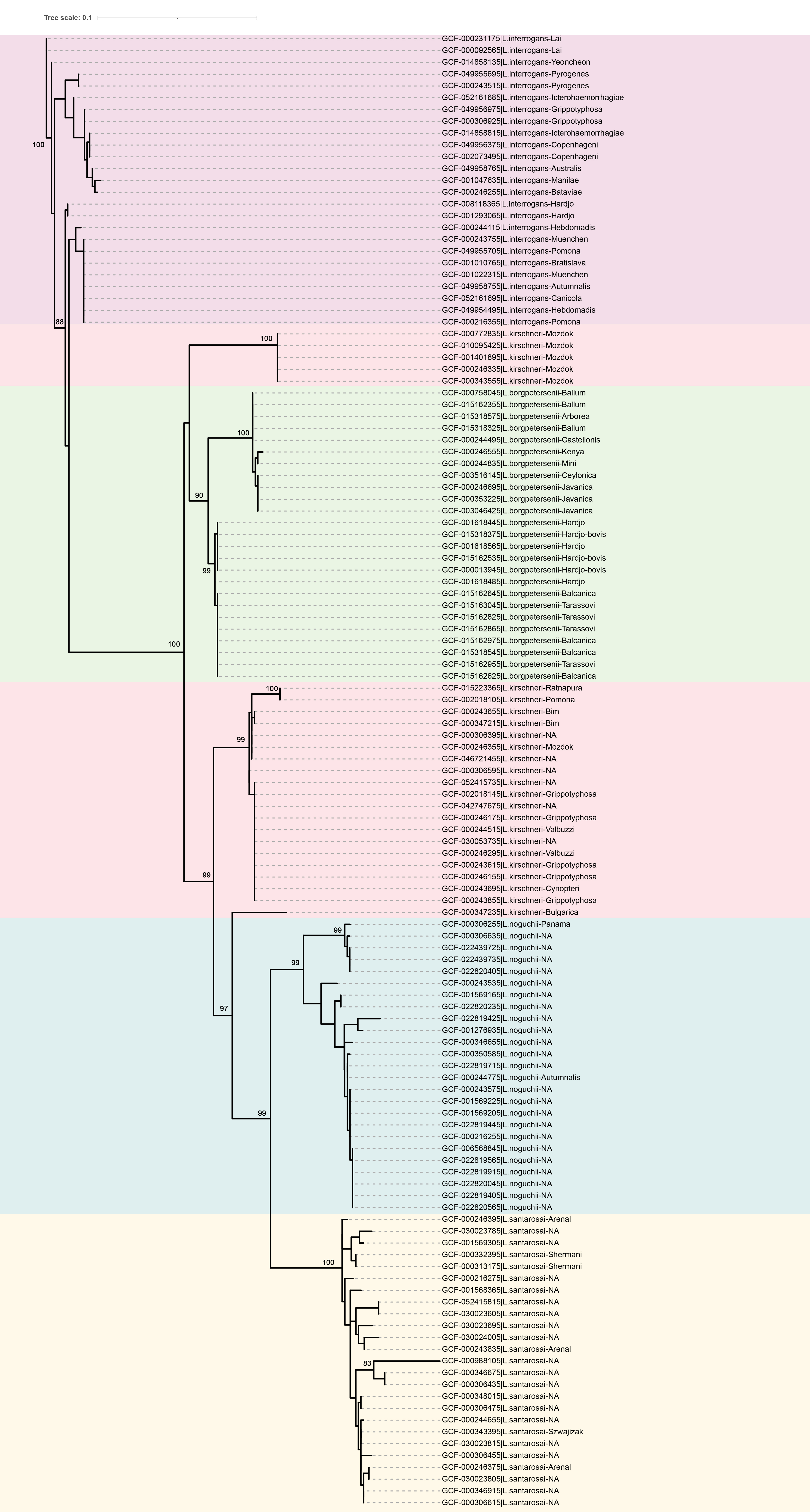

Supplement: Supplementary file 4 [file Image_4.JPEG]

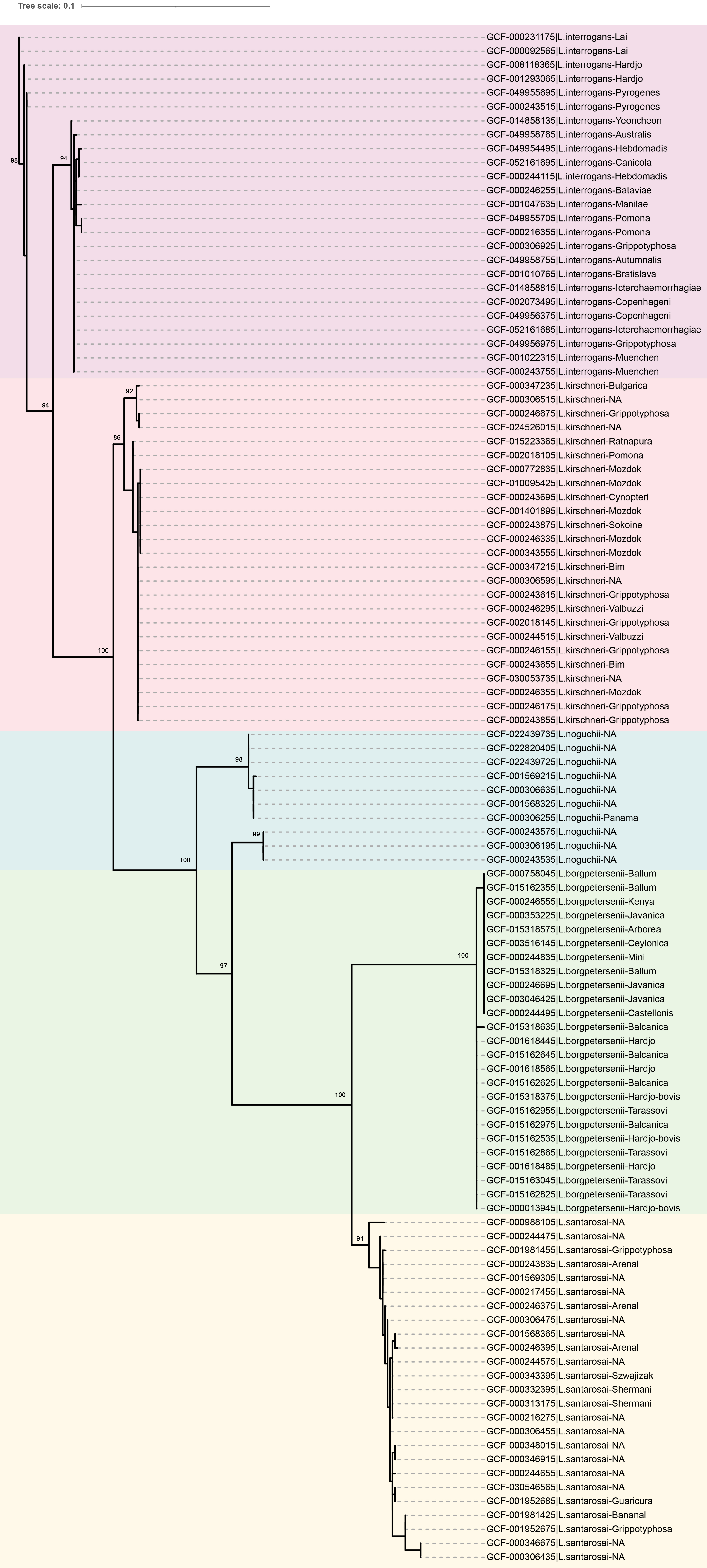

Supplement: Supplementary file 5 [file Image_5.JPEG]

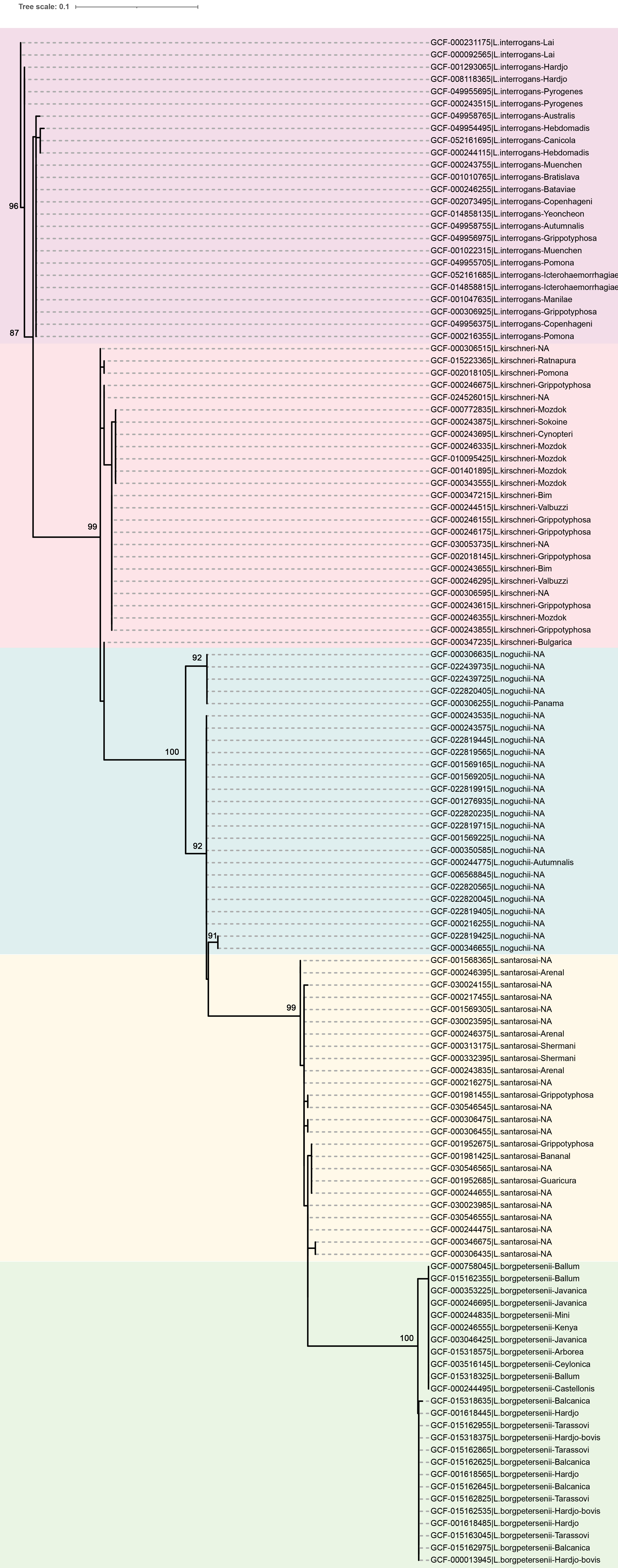

Supplement: Supplementary file 6 [file Image_6.JPEG]

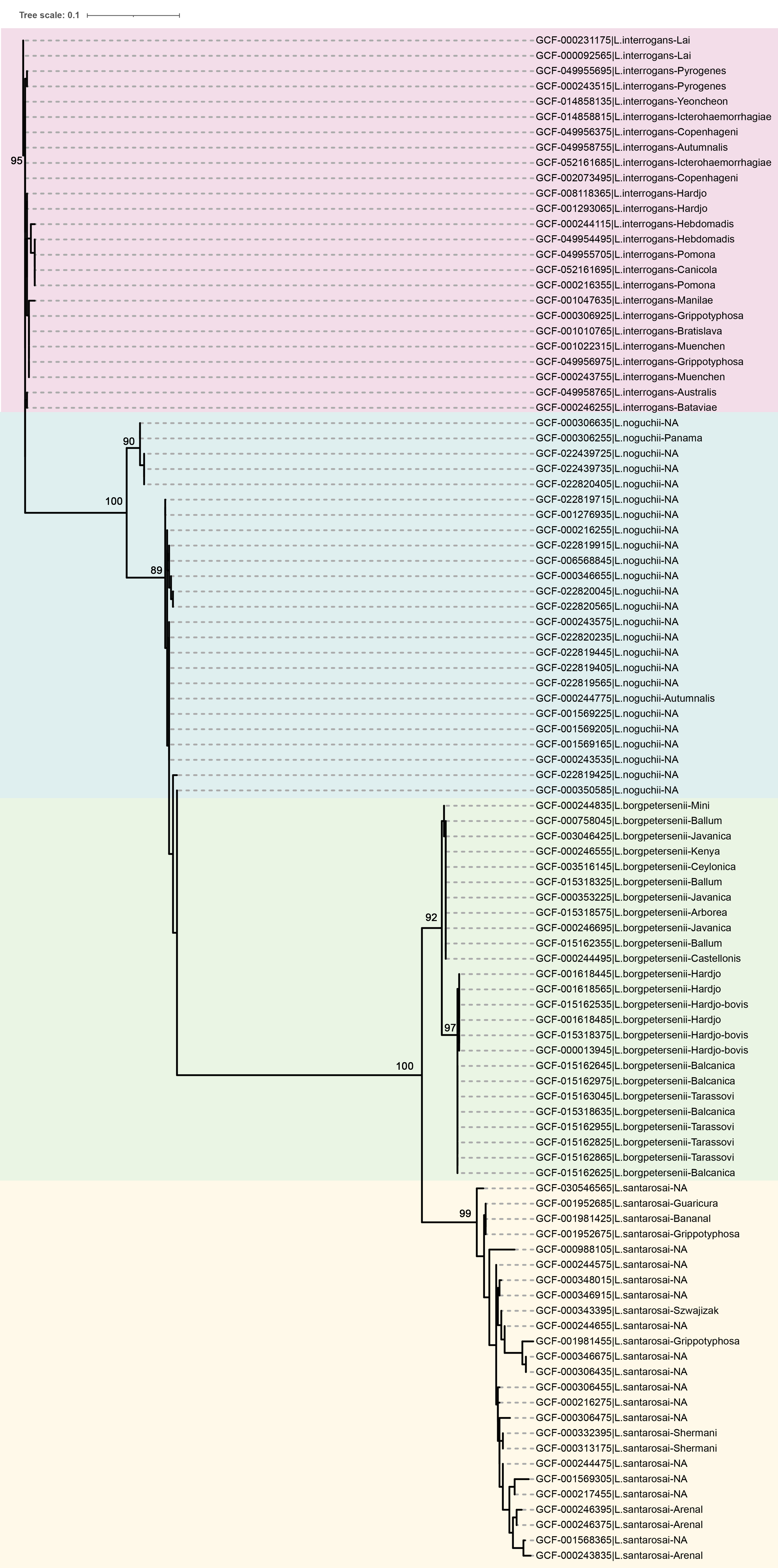

Supplement: Supplementary file 7 [file Image_7.JPEG]
